# Effect of degraded flaxseed meals on the growth performance, nutrient digestibility, and health status of broilers

**DOI:** 10.5713/ab.23.0416

**Published:** 2024-02-23

**Authors:** Xiaoyu Ji, Xiangyu Liu, Jianping Wang, Ning Liu

**Affiliations:** 1 Department of Animal Science, Henan University of Science and Technology, Luoyang 471000, China

**Keywords:** Broiler, Enzymolysis, Fermentation, Flaxseed Meal, Nutritive Value

## Abstract

**Objective:**

The present study evaluated the effect of flaxseed meal degraded by a protease, *Lactobacillus plantarum*, or both on the growth performance, nutrient digestibility, and health status of broilers.

**Methods:**

There were four diets containing flaxseed meals in its non-degraded form (control, CON), degraded with 3,000 U/kg of protease (enzymatic, ELM), 1.0×10^9^ CFU/kg of *Lactobacillus plantarum* (fermented, FLM), or both (dual-degraded, DLM). Each form of flaxseed meals was added at 15% of diet. A total of 480 yellow-feathered broilers at 22 d of age were distributed into 4 groups with 6 replicates of 20 chickens each. The feeding trial lasted for 42 d. Growth performance, apparent fecal digestibility (dry matter, energy, crude protein, and ash), and serum immunoglobins and antioxidases were determined at 42 and 63 d of age.

**Results:**

Results showed that ELM, FLM, and DLM increased (p<0.001) the contents of peptides and decreased (p<0.001) cyanogenic glycosides, compared to CON. The diets with degraded flaxseed meals increased (p<0.05) feed intake and body weight gain throughout the feeding trial, and the digestibility of energy, crude protein, and ash at the end of feeding trial. Furthermore, all degraded groups enhanced (p<0.05) broiler health status by increasing serum immunoglobulins A and G. Additinally, DLM showed more pronounced effects (p<0.05) on these parameters than ELM or FLM.

**Conclusion:**

Flaxseed meals degraded by enzymolysis, fermentation, or both had improved nutrition and application in broilers.

## INTRODUCTION

Increased uncertainty in the global food supply has led to shortages in protein-sourced feedstuffs, which is a huge challenge to the animal production. Protein improvement of feedstuffs is being investigated in an effort to relieve the shortage. Flax (*Linum usitatissimum* L.), a crop for fiber and oil production, is native to Asia, Europe, and Mediterranean. Flaxseed meal is obtained after flaxseed oil extraction, containing approximately 34% of protein, 1.8% of fat, and 8% of fiber [1]. Additionally, it contains beneficial phytoactives, such as phytoestrogens and α-linolenic acid, and also negative compounds, including cyanogenic glycosides, trypsin inhibitors, and phytic acid [1]. Based on a high protein content and availability, the flaxseed meal is now listed as a protein feedstuff in the feed database of many countries [2,3]. A study showed that flaxseed meal can replace 50% of soybean meal in the diet without adverse effects on the growth and health of broilers [4] and extruded flaxseed at 27% of diet enriched egg n-3 fatty acids [5].

The technlologies involved in protein improvement of feedstuffs mainly include enzyme hydrolysis and fermentation. Pretreatment with enzymes or microbes can degrade some whole protein molecules into peptides which enhances nitrogen bioavailability [6]. Additionally, some poisonous or antinutritional factors can also be inactivated to some extent in the pretreatment process. Cyanogenic glycosides and phytic acid are major concerns in flaxseed meal [7]. The former can be greatly reduced with a hot-extrusion in the process of oil production and can be further diminished with fermentation [8]. For the latter, as known, the use of enzymes or fermentation has a better decomposition. In practice, a single fermentation method does not usually fulfill actual requirements, whereas co-treatment with microbes and enzymes is more likely to improve feed utilization and to reduce anti-nutrients [6]. Several researches had found that fermentation can enhance the nutritional value and utilization of flaxseed meals in the diets of pigs [9,10] and poultry [11]. However, literature is unavailable about the pretreatmeat of flaxseed meals with enzymatic degradation.

Therefore, the present study aimed to evaluate the effects of protease, probiotic, or both on the nutritional changes of flaxseed meals and their application in broiler production.

## MATERIALS AND METHODS

### Animal care

Research on animals was conducted according to the Animal Welfare Committee of Henan University of Science and Technology on animal use (No. 2021011).

### Experimental diets

Flaxseed meal is a by-product of flaxseed kernels after oil extraction with heating detoxification at 50°C for 2 h containing cyanogenic glycosides of 93.6 mg/kg. Flaxseed meal was ground (16 mesh size) and sterilized at 121°C for 20 min. For solid-state fermentation, sterilized flaxseed meal and deionized water were mixed at 100:35.

Enzyme PROMAX Protease (pH 1.5 to 6.0) was supplied by Challenge International Trade Co. (Beijing, China). For enzymatic hydrolysis of flaxseed meal, substrate was mixed with the protease at the activity of 3,000 U/kg at 28°C for 48 h. *Lactobacillus plantarum* M2016136 (*L. plantarum*) was supplied by China General Microbiological Culture Collection Center (CCTCC, Wuhan, China). *L. plantarum* was incubated at 32°C for 48 h with 1.0×10^9^ CFU/kg fermented substrate to produce fermented flaxseed meal. The protease and *L. plantarum* were incubated with fermented substrates at 32°C for 48 h to prepare a double-treated flaxseed meal. The freeze-dried and ground products of enzymolysis, fermentation, or both were used to prepare the four diets (16 mesh size). Compositional differences of flaxseed meals degraded by the protease, the probiotic or both are listed in Table 1.

The non-degraded (control, CON), enzymatic (ELM), fermented (FLM), and dual-degraded (DLM) flaxseed meals were used as a protein source of four diets, and each of them was added to 15%. The diets were established according to the Nutritional Requirement of Chinese Yellow-feathered Broilers (Standards in Agricultural Industries in China, NY/T 3645-2020; Table 2).

### Animals and samples

A total of 480 female yellow-feathered broilers at 18 d of age with similar body weight (415±4.53 g, BW±standard deviation) were randomly distributed into four groups, with six replicates of 20 broilers each (pens). A three-day adjustment was made using the control diet. The feeding trial lasted from 22 to 63 d of age with two phases, 22 to 42 and 43 to 63 d. During the feeding trial, all broilers had free access to diets and water. The chicken house was set at lighting time of 23 h/d, humidity of 50% to 60%, 20°C, and automatic mechanical ventilation. At 21, 42, and 63 d, broilers and feed were weighed, and feed efficiency was adjusted on a replicate basis. Health status of broilers was monitored twice a day.

At 42 and 63 d, ten broilers per replicate were randomly selected for blood collection from the wing veins. Serum samples were prepared by centrifuging the blood at 3,000 g at 4°C for 10 min and stored at −20°C for the analysis of immunoglobulins and antioxidant enzymes. Excreta samples were collected at 40 to 42 and 61 to 63 d of age to determine the apparent digestibility of dry matter, energy, crude protein, and ash.

### Chemical analysis

The contents of nutrients in the feed and feces were determined according to AOAC (1990) for total P (964.06), phytate P (927.02), Ca (935.13), crude protein (976.05), crude fat (920.39), crude fiber (962.09), and ash (942.05). Other parameters included gross energy (Oxygen Bomb Calorimeter, Model 6300; PARR, Moline, IL, USA), metabolizable energy (ME) by the method of adult roosters [12], dry matter by drying a 2 g sample at 105°C to a constant mass, amino acids (AA analyzer, Beckman 6300; Beckman Coulter, Inc., Fullerton, CA, USA), acid-soluble protein (The Standards in Agricultural Industries in China NY/T 3801-2020), and titanium dioxide as an inert marker [13].

Cyanogenic glycosides were determined by the colorimetric method according to The China National Standard GB/T 13084-2006 (Determination of Cyanide in Feed) with detection limits of 0.01 g/mL. Briefly, one mL of 10 g/L sodium hydroxide solution and 1 drop of phenolphthalein indicator solution were added to the sample solution and slowly adjusted with the acetic acid solution until the red color faded. Then, 5 mL of phosphate solution were added and held for 10 min at 37°C in a constant temperature water bath; and then, 0.25 mL of chloramine T solution were added, mixed well and kept still for 5 min. At last, 5 mL of isonicotinic acid-pyrazolone solution and water were added to the final volume of 25 mL, and mixed well. The absorbance was measured at 638 nm using a colorimeter (722s; Shanghai Precision Science Instrument Co., Shanghai, China).

Peptides (≤1 kDa) were quantified using electrophoresis in sodium dodecyl sulfate-polyacrylamide gels [14]. For organic acids estimation, the sample slurry was centrifuged at 1×10^4^ g for 20 min. The supernatant was analyzed using HPLC (Model 1100; Agilent Technologies, Inc, Santa Clara, CA, USA) with 10 mM perchloric acid as a mobile phase at 40°C.

### Biochemical analysis and bacterial enumeration

Protease activity was analyzed according to The China National Standard GB/T 28715-2012. A protease activity unit is the enzyme amount that hydrolyzes casein to produce color equivalent to 1.0 μmole of tyrosine per minute at 37°C, pH 7.5. Commercial kits from Nanjing Jiancheng Biological Institutes (Nanjing, China) were used for detecting superoxide dismutase (A001-0302) and glutathione peroxidase (A005-102). Concentrations of immunoglobulin A (IgA) and IgG in the serum were measured using an automatic biochemistry analyzer (Model 7600; Hitachi High-Tech, Tokyo, Japan).

*L. plantarum* was enumerated according to China National Standard GB/T 26428-2010 using a nutrient agar base (No. HB0385-1; Hopebio, Qingdao, China) at 36°C, pH 7.0, for 24 h.

### Statistics

Data were expressed as mean and standard error of the mean using the analysis of variance (ANOVA) general linear model procedure (IBM SPSS, Armonk, NY, USA). The statistical unit was all birds per replicate for growth performance and nutrient digestibility, the mean of ten birds for blood parameters. Tukey’s-b test (homoscedasticity assumption) or Tamhane T2 (heteroskedasticity assumption) were used to separate differences in ANOVA variables at a significance level of p<0.05.

## RESULTS

### Compositions of flaxseed meal degraded by protease, probiotic, or both

Flaxseed meal degraded with protease, *L. plantarum*, or both increased (p≤0.043) acid-soluble protein, polypeptides, non-phytate P, and organic acid, but decreased (p≤0.027) nitrogen-free extract, crude fiber, and cyanogenic glycoside (Table 1). The degradation of flaxseed meals did not affect amino acids, total thiol-containing amino acids, and total necessary amino acids (data not shown). Polypeptide content in DLM was higher (p<0.05) than that in ELM and FLM. Also, the diets with fermentation treatment contained higher (p<0.05) organic acids. Nitrogen-free extract in FLM and DLM was decreased (p<0.05), compared to CON and ELM. DLM showed the lowest (p<0.05) content of cyanogenic glycoside among treatments.

### Growth performance of broilers

During 21–42 d of age, ELM, FLM, and DLM increased (p = 0.036) feed intake, compared to CON; DLM showed greater (p<0.05) BW gain than CON and FLM (Table 3). During 21–63 d of age, diets with degraded flaxseed meals increased (p≤0.011) feed intake and BW gain; feed/gain in ELM was lower (p<0.05) than CON and FLM. There were no differences in mortality among groups throughout the feeding trial.

### Apparent digestibility of nutrients

Compared to CON, all degraded groups increased (p≤0.042) the dry matter, energy, crude protein, and ash during 40–42 d and 61–63 d of age (Table 4). During 40–42 d of age, the digestibility of crude protein in ELM and DLM was higher (p<0.05) than FLM. During 61–63 d of age, the apparent digestibility of dry matter and crude protein in DLM was higher (p<0.05) than FLM.

### Serum immunoglobulins and anti-oxidases

At 42 d, the degraded groups increased (p≤0.030) the levels of IgG, IgA, superoxide dismutase, and glutathione peroxidase, compared to CON (Table 5), but there were no differences among the three degraded groups. At 63 d, all diets with degraded flaxseed meals increased (p≤0.037) IgG and IgA; DLM showed a higher (p<0.05) activity of glutathione peroxidase than CON; ELM had higher (p<0.05) IgG and IgA than FLM; and IgA in FLM was lower (p<0.05) than ELM and DLM.

## DISCUSSION

In the present study, degradation by enzymolysis, fermentation, or both improved the nutritional compositions of flaxseed meals and reduced cyanogenic glycoside content; polypeptides and organic acids were significantly increased in the groups with fermented flaxseed meals; and DLM had the best effects on these parameters. Thanabalan et al [15] found that the content of organic acid in full-fat flaxseed diets was not affected by the presence of multienzymes (galactanase, protease, mannanase, dextranase, xylanase, amylase, and cellulase. The enzymolysis in ELM did not affect crude protein and dry matter; and FLM and DLM increased crude protein content. However, literature reported that fermentation increased crude protein content of flaxseed cake in duckling diets [11]. During the fermentation process, energy substances such as sugars are preferentially utilized for microbial growth, resulting in high crude protein content after fermentation.

Flaxseed meal is a source of crude protein, but its use in animal feed is limited due to a high cyanogenic glycoside content [1]. FLM and DLM groups had decreased crude fiber and cyanogenic glycosides, which may resulted from fermentation degradation. Indeed, studies showed that fermentation decreased crude fiber and cyanogenic glycosides in duck diets [11,16]. FLM and DLM had lower nitrogen-free extract, which may be ascribed to fermentation depletion of carbohydrates. Flaxseed peptides as a feed ingredient have not been widely studied, and more research is required. Additionally, enzyme addition reduced arachidonic acid and total long-chain n-6 fatty acids but enhanced long-chain n-3 fatty acids in hepatic phospholipids [17]. Therefore, the effect of fermentation on the fatty acids of flaxseed meal deserves further study.

A few studies reported flaxseed meal as a partial replacement of soybean meal on the growth performance of farm animals [2,4,9], but the information is limited about *in vitro* pretreatment or dietary co-administration with enzymes and probiotics. Apperson and Cherian [18] found that 15% flaxseed with carbohydrases had no effect on BW, average daily gain, and feed intake. Head et al [17] observed similar results in a diet of 10% flaxseed meal treated with 0.05% carbohydrases. Jia and Slominski [19] showed that 15% full-fat flaxseed with enzymes (cellulase, pectinase, xylanase/glycanase) did not affect the growth performance of broilers. Fish fed diets supplemented with probiotics (4×10^9^ CFU/kg) had the highest carcass composition [20]. In contrast, in the present study, broilers fed on ELM, FLM, or DLM had increased feed intake and BW, possibly due to the reduced toxins, and increased peptides and oranic acids. Furthermore, DLM has a significant effect on growth performance.

In the present study, the diets containing degraded meals increased the digestibility of dry matter, crude protein, energy, and ash. Saleh et al [21] reported that flaxseed meals with enzymes (xylanase, cellulase, β-mannanase, phytase, α-amylase, and protease) improved the digestibility of crude protein and crude fiber in broilers. This may partially explain the beneficial effect of flaxseed peptides on broiler growth. Thanabalan et al [15] reported that a full-fat flaxseed diet supplemented with multienzymes retained less dry matter and crude protein and improved apparent metabolizable energy (AME) retention. Slominski et al [22] found that multienzymes (cellulase, pectinase, and xylanase) improved nitrogen-corrected apparent metabolizable enrgy (AMEn) content of full-fat flaxseed in adult roosters. The AME in the present study was non-significantly decreased in ELM, FLM, and DLM. It is worth noting that the differences in AME between literature and the present study may be caused by the different forms of flaxseed, such as whole seed, ground seed, and heat-treated seed, which warrants further investigation.

The degraded flaxseed meals in the present study increased serum immunoglobulin concentrations and antioxidant enzyme activity, indicating that some compounds such as flaxseed peptides and organic acids can improve the antioxidant capacity and immune function of broilers. Literature about this is unavailable, and more research is needed on the effects of flaxseed peptides on antioxidant capacity and immunity in animals. Besides these, the lower phytic acid and cyanogenic glycosides in the degraded diets may also contribute to the health improvement of broilers. Additionaly, there may be a beneficial effect of flaxseed oil on the immunity of animals due to the presence of α-linolenic acid and its derivatives [23] although the oil is very low in the flaxseed meal. Zając et al [24] reported that a flaxseed diet reduced hemoglobin levels, which may be related to the levels of cyanogenic glycosides (linamarins, linustatins, and neolinustatin) and enzymes (β-bis-glucosidase, β-monoglucosidase, and α-hydroxy nitrile lyase) involved in the hydrolysis of cyanogenic glycosides and release of hydrocyanic acid. Therefore, studies on antioxidation, immunity, and antimicrobial properties deserve further investigation. Additionally, in practice, a better effect on the quality improvement of flaxseed meals can be found by the treatment with a selected mixture of enzymes and microbes.

## CONCLUSION

Flaxseed meals pretreated with protease, probiotic, or both can improve the nutritional composition, digestibility, and health of broilers. Dual degradation has a greater impact on these parameters. It is recommended to treat a feed with enzymes and probiotics simultaneously to advance feed development and utilization.

## Figures and Tables

**Table 1:** Compositional differences of flaxseed meal degraded by protease, probiotic or both (dry matter basis)

Item	CON^1)^	Degraded			SEM	p-value

		ELM^1)^	FLM^1)^	DLM^1)^		
Protein, peptides, and amino acids (%)						
Crude protein	34.8^b^	34.9^b^	36.3^a^	36.6^a^	0.036	0.043
Acid-soluble protein	4.02^d^	21.7^b^	9.86^c^	25.8^a^	0.326	<0.001
Polypeptides (≤1 kDa)	5.01^d^	39.6^b^	27.5^c^	46.9^a^	1.271	<0.001
Lysine	1.16	1.21	1.11	1.08	0.092	0.437
Methionine	0.56	0.53	0.57	0.58	0.011	0.262
Thiol amino acid	1.11	1.09	1.23	1.22	0.139	0.654
Carbohydrates, lipids, and minerals (%)						
Nitrogen-free extract	36.7^a^	34.2^a^	19.3^b^	18.9^b^	0.951	<0.001
Crude fiber	8.22^a^	8.54^a^	5.72^b^	6.28^b^	0.478	0.027
Ether extract	1.85	1.76	2.19	2.04	0.167	0.252
Crude ash	6.31	6.58	6.92	6.81	0.429	0.973
Total P	0.96	0.92	0.98	1.01	0.122	0.876
Non-phytate P	0.25^b^	0.24^b^	0.47^a^	0.56^a^	0.293	<0.001
Energy values (MJ/kg) and others (%)						
Gross energy	15.7	16.1	14.8	14.0	0.986	0.088
Metabolizable energy	10.1	9.86	9.91	9.69	0.357	0.102
Dry matter	87.8	88.1	87.9	88.2	0.269	0.896
Organic acid	0.47^b^	0.52^b^	4.35^a^	4.52^a^	0.274	<0.001
Protease (U/kg)	-	342^a^	61.9^b^	411^a^	27.08	<0.001
*L. plantarum* (Log_10_ cfu/kg)	-	-	3.11	2.98	0.275	0.672
Cyanogenic glycoside (mg/kg)	93.6^a^	35.5^b^	37.6^b^	22.8^c^	3.069	<0.001

SEM, standard error of the mean.

1) CON, non-degraded flaxseed meal; ELM, flaxseed meal was degraded with enzyme PROMAX Protease (pH 1.5 to 6.0; Challenge International Trade Co., Beijing, China); FLM, flaxseed meal was fermented with *Lactobacillus plantarum* (No. CCTCC M2016136; China Center for Type Culture Collection, Wuhan, China); DLM, flaxseed meal was dual-degraded with the protease and the probiotic.

a–d Means among treatments without the same superscript are significantly different (p<0.05).

**Table 2:** Ingredients and chemical compositions of diets (air-dry basis) for yellow-feathered broilers

Ingredients (%) at 22–42/43–63 d				
Flaxseed meal	15/15			
Corn gluten	12.7/10.5			
Corn	65.0/66.8			
Soybean oil	2.0/3.0			
Lysine	0.7/0.6			
Methionine	0.2/0.2			
Limestone	1.3/1.4			
Dicalcium phosphate	1.7/1.1			
Sodium chloride	0.4/0.4			
Titanium dioxide	0.5/0.5			
Premix^1)^	0.5/0.5			

**Chemical compositions (%) at 22–42/43–63 d**	**CON** ^2)^	**ELM** ^2)^	**FLM** ^2)^	**DLM** ^2)^

Calculated nutrients				
Metabolizable energy (MJ/kg)^3)^	12.79/13.19	12.75/13.15	12.76/13.16	12.73/13.13
Determined nutrients				
Crude protein	19.38/18.03	19.40/18.05	19.61/18.26	19.65/18.30
Calcium	0.94/0.84	0.94/0.84	0.94/0.84	0.94/0.84
Non-phytate P	0.42/0.36	0.42/0.36	0.45/0.36	0.47/0.36
Lysine	1.17/1.02	1.18/1.01	1.16/1.03	1.16/1.04
Methionine	0.60/0.52	0.59/0.52	0.60/0.52	0.60/0.57
Methionine+cysteine	0.83/0.74	0.83/0.74	0.84/0.74	0.84/0.79

1) Supplied per kilogram of diet for 22–42/43–63 d: vitamin A 9,000/6,000 IU, vitamin D_3_ 500/500 IU, vitamin E 35/25 mg, vitamin K 2.2/1.7 mg, thiamin 2.3/1.0 mg, riboflavin 5.0/4.0 mg, niacin 35/20 mg, pantothenic acid 10/8 mg, pyridoxine 2.4/0.6 mg, biotin 0.10/0.02 mg, folic acid 0.7/0.3 mg, Vitamin B_12_ 1.0/0.75 mg, K 4.6/4.0 g, Mg 0.6/0.6 g, Fe 80/80 mg, Cu 7/7 mg, Mn 60/55 mg, Zn 80/75 mg, I 0.6/0.5 mg, Se 0.15/0.15 mg.

2) CON, containing non-degraded flaxseed meal at 15%; ELM, containing 15% flaxseed meal degraded with enzyme PROMAX Protease (pH 1.5 to 6.0; Challenge International Trade Co., Beijing, China); FLM, containing 15% flaxseed meal fermented with *Lactobacillus plantarum* (No. CCTCC M2016136; China Center for Type Culture Collection, Wuhan, China); DLM, containing flaxseed meal 15% dual-degraded with the protease and the probiotic.

3) Calculated according to China Feed Data (Xiong et al [3]).

**Table 3:** Effect of flaxseed meal fermented with enzyme addition on the growth performance of yellow-feathered broilers

Items	CON^1)^	Degraded			SEM	p-value

		ELM^1)^	FLM^1)^	DLM^1)^		
22 to 42 d of age						
Initial body weight (g/bird)	414	417	417	414	1.849	0.752
Final body weight (kg/bird)	1.27^b^	1.29^b^	1.28^b^	1.32^a^	0.009	0.013
Feed intake (kg/bird)	1.97^b^	2.07^a^	2.04^a^	2.05^a^	0.015	0.036
Body weight gain (g/bird)	855^b^	877^ab^	869^b^	908^a^	9.877	0.017
Feed/gain	2.30	2.36	2.35	2.26	0.029	0.195
Mortality (%)	2.50	1.67	2.50	1.67	1.086	0.898
22 to 63 d of age						
Final body weight (kg/bird)	1.78^b^	2.03^a^	1.93^a^	2.03^a^	0.049	0.026
Feed intake (kg/bird)	4.21^b^	4.40^a^	4.43^a^	4.62^a^	0.086	0.011
Body weight gain (kg/bird)	1.37^b^	1.61^a^	1.51^a^	1.62^a^	0.018	0.007
Feed/gain (kg/kg)	3.07^a^	2.73^b^	2.93^a^	2.85^ab^	0.027	0.031
Mortality (%)	2.50	3.33	2.50	1.67	1.015	0.724

1) CON, containing non-degraded flaxseed meal at 15%; ELM, containing 15% flaxseed meal degraded with enzyme PROMAX Protease (pH 1.5 to 6.0; Challenge International Trade Co., Beijing, China); FLM, containing 15% flaxseed meal fermented with *Lactobacillus plantarum* (No. CCTCC M2016136; China Center for Type Culture Collection, Wuhan, China); DLM, containing flaxseed meal 15% dual-degraded with the protease and the probiotic.

a,b Means among treatments without the same superscript are significantly different (p<0.05).

**Table 4:** Effect of flaxseed meal fermented with enzyme addition on the apparent digestibility of nutrients in yellow-feathered broilers

Item	CON^1)^	Degraded			SEM	p-value

		ELM^1)^	FLM^1)^	DLM^1)^		
40 to 42 d of age (%)						
Dry matter	72.2^b^	76.9^a^	74.5^ab^	77.3^a^	1.015	0.042
Energy	67.8^c^	74.2^a^	75.1^a^	75.6^a^	1.116	0.039
Crude protein	71.7^c^	77.8^a^	75.4^b^	78.8^a^	1.012	0.011
Ash	43.6^b^	48.5^a^	50.2^a^	52.7^a^	1.835	0.026
61 to 63 d of age (%)						
Dry matter	69.2^c^	73.1^ab^	72.9^b^	75.6^a^	0.784	0.006
Energy	65.8^b^	71.4^a^	70.5^a^	72.6^a^	1.310	0.046
Crude protein	69.5^c^	74.6^a^	71.7^b^	75.1^a^	1.442	0.027
Ash	37.2^b^	43.8^a^	44.2^a^	44.7^a^	1.336	0.029

1) CON, containing non-degraded flaxseed meal at 15%; ELM, containing 15% flaxseed meal degraded with enzyme PROMAX Protease (pH 1.5 to 6.0; Challenge International Trade Co., Beijing, China); FLM, containing 15% flaxseed meal fermented with *Lactobacillus plantarum* (No. CCTCC M2016136; China Center for Type Culture Collection, Wuhan, China); DLM, containing flaxseed meal 15% dual-degraded with the protease and the probiotic.

a–c Means among treatments without the same superscript are significantly different (p<0.05).

**Table 5:** Effect of flaxseed meal fermented with enzyme addition on serum immunoglobulins and antioxidative enzymes of broilers

Item	CON^1)^	Degraded			SEM	p-value

		ELM^1)^	FLM^1)^	DLM^1)^		
42 d of age						
IgG (g/L)	0.23^b^	0.28^a^	0.29^a^	0.30^a^	0.009	0.012
IgA (g/L)	0.17^b^	0.22^a^	0.20^a^	0.25^a^	0.018	0.022
Superoxide dismutase (U/mL)	27.4^b^	33.8^a^	33.1^a^	36.5^a^	1.326	0.030
Glutathione peroxidase (U/mL)	18.6^b^	25.2^a^	23.7^a^	24.8^a^	0.784	0.006
63 d of age						
IgG (g/L)	0.22^b^	0.26^a^	0.22^b^	0.25^ab^	0.009	0.042
IgA (g/L)	0.18^b^	0.24^a^	0.21^b^	0.26^a^	0.006	0.021
Superoxide dismutase (U/mL)	25.8	27.6	28.2	28.5	0.023	0.086
Glutathione peroxidase (U/mL)	17.5^b^	19.6^ab^	19.3^ab^	22.2^a^	0.812	0.037

SEM, standard error of the mean; IgG, immunoglobulin G.

1) CON, containing non-degraded flaxseed meal at 15%; ELM, containing 15% flaxseed meal degraded with enzyme PROMAX Protease (pH 1.5 to 6.0; Challenge International Trade Co., Beijing, China); FLM, containing 15% flaxseed meal fermented with *Lactobacillus plantarum* (No. CCTCC M2016136; China Center for Type Culture Collection, Wuhan, China); DLM, containing flaxseed meal 15% dual-degraded with the protease and the probiotic.

a,b Means within a row not sharing a superscript are significantly different (p<0.05).
